# Association between triglyceride–glucose index and diabetic retinopathy among patients with diabetes mellitus in Nepalese patients: a cross-sectional study

**DOI:** 10.1186/s12902-026-02191-4

**Published:** 2026-02-07

**Authors:** Saurav Thapa, Anup Ghimire, Bijay Kunwar, Binay Aryal, Aramva Bikram Adhikari, Arati Karakheti, Mani Prasad Gautam

**Affiliations:** 1 Department of Internal Medicine, National Academy of Medical Sciences, Bir Hospital, Kathmandu, Nepal; 2 Tribhuvan University Institute of Medicine, Maharajgunj Medical Campus, P.O. Box: 1524, Kathmandu-3, Kathmandu District, Kathmandu, Bagmati Nepal; 3 Department of Ophthalmology, National Academy of Medical Sciences, Bir Hospital, Kathmandu, Nepal

**Keywords:** Triglyceride glucose index, Diabetic retinopathy, Diabetes mellitus

## Abstract

**Background:**

Diabetic retinopathy (DR) is one of the most important microvascular complications of diabetes mellitus and remains a major cause of preventable blindness worldwide. Insulin resistance plays a pivotal role in the development of diabetes and its associated complications. The triglyceride–glucose (TyG) index, a surrogate marker of insulin resistance derived from fasting plasma glucose and triglyceride levels, has gained attention as a simple and practical metabolic marker. However, data linking the TyG index with diabetic retinopathy, particularly in South Asian populations, are still limited.

**Methods:**

A hospital-based cross-sectional study was conducted at Bir Hospital, Kathmandu, Nepal, between April 2023 and October 2024. Adult patients with diabetes mellitus, excluding those with gestational diabetes, acute illness, non-diabetic causes of hypertriglyceridaemia, conditions affecting lipid or glucose metabolism, prior retinal interventions, or non-diabetic retinal diseases, underwent comprehensive clinical, biochemical, and ophthalmic evaluations. The triglyceride–glucose (TyG) index was calculated as ln [fasting triglycerides (mg/dL) × fasting plasma glucose (mg/dL) / 2], and participants were categorized into quartiles. Diabetic retinopathy was graded using internationally accepted clinical severity scales. Associations were assessed using univariate and multivariate logistic regression, and ROC curve analysis evaluated the discriminatory performance of the TyG index.

**Results:**

Among 83 patients with diabetes mellitus (median age 56 years [IQR 48.5–64], 50.6% male, median diabetes duration 6 years [IQR 3–12]), the median triglyceride-glucose (TyG) index was 9.98 (IQR 9.485–10.445). Diabetic retinopathy (DR) prevalence was 48.2%, increasing across TyG quartiles from 9.5% in Q1 to 85.7% in Q4 (*p* < 0.001), with a significant trend in DR severity (*p* < 0.001). TyG correlated moderately with HbA1c (Spearman *r* = 0.54, *p* < 0.001). In multivariable logistic regression adjusted for age, total cholesterol, and HDL, the odds ratio for DR per standard deviation increase in TyG was 4.00 (95% CI 1.75–9.16, *p* = 0.001); for Q4 vs. Q1, 24.15 (95% CI 3.15–185, *p* = 0.002). Findings were robust in sensitivity analyses excluding insulin users (OR 4.35, 95% CI 1.40–13.5, *p* = 0.011) and outliers (OR 3.80, 95% CI 1.60-9.00, *p* = 0.002), and stratified by diabetes duration (≥ 5 years: OR 5.20, 95% CI 1.80–15.0, *p* = 0.002) and sex (males: OR 3.80, 95% CI 1.20–12.0, *p* = 0.023; females: OR 4.50, 95% CI 1.30–15.5, *p* = 0.017). TyG predicted DR with an area under the ROC curve of 0.83 (95% CI 0.75–0.91), comparable to HbA1c (0.77, DeLong *p* = 0.29); optimal cutoff 9.78 (sensitivity 88%, specificity 67%).

**Conclusions:**

A higher triglyceride–glucose index was strongly associated with both the presence and severity of diabetic retinopathy. Given its simplicity and reliance on routinely available laboratory parameters, the TyG index may be a useful marker for identifying diabetic patients at increased risk of retinopathy, particularly in resource-limited settings.

**Clinical trial registration:**

Not applicable. This was a cross-sectional observational study and did not involve a clinical trial requiring registration.

**Supplementary Information:**

The online version contains supplementary material available at 10.1186/s12902-026-02191-4.

## Introduction

 Diabetic retinopathy (DR) is among the most common microvascular complications of diabetes mellitus and continues to be a leading cause of preventable visual impairment worldwide. Meta-analytic evidence indicates that nearly one-third of individuals with diabetes develop some degree of diabetic retinopathy, with a substantial proportion progressing to vision-threatening stages [1]. The burden of DR is rising rapidly in low- and middle-income countries, in parallel with the increasing prevalence of diabetes mellitus [2]. In Nepal, the prevalence of diabetes has increased markedly over recent decades, and diabetic retinopathy is now emerging as a significant public health concern [3, 4].

Insulin resistance is a key pathogenic mechanism in type 2 diabetes mellitus and contributes to both macrovascular and microvascular complications. Persistent hyperglycaemia and dyslipidaemia promote oxidative stress, endothelial dysfunction, inflammation, and microvascular injury, all of which play important roles in the development and progression of diabetic retinopathy [5–7]. Identifying simple and reliable markers of insulin resistance may therefore be valuable for early risk stratification of diabetic complications.

The triglyceride–glucose (TyG) index, calculated using fasting plasma glucose and triglyceride levels, has been validated as a surrogate marker of insulin resistance and shows good correlation with the hyperinsulinaemic–euglycaemic clamp technique and HOMA-IR [8–10]. Because it relies on routinely available laboratory tests, the TyG index is particularly appealing for use in resource-limited settings.

Emerging evidence suggests that the TyG index is associated with cardiovascular disease, metabolic syndrome, and diabetic microvascular complications [11–14]. Several recent studies have reported an association between the TyG index and diabetic retinopathy; however, most of the available data are derived from East Asian populations, and evidence from South Asia remains scarce [15–17]. This study was therefore conducted to evaluate the diagnostic performance and threshold value of the TyG index as a screening tool for DR in a clinical setting and severity of diabetic retinopathy among patients attending a tertiary care hospital in Nepal.

## Methods

### Study design and setting

This hospital-based cross-sectional observational study was conducted at Bir Hospital, Kathmandu, Nepal, from April 2023 to October 2024. In accordance with the STROBE guidelines for cross-sectional studies, the study size was determined a priori based on the primary objective of estimating the prevalence of diabetic retinopathy in the study population. Since the study aimed to estimate the prevalence of diabetic retinopathy, a binary outcome, the sample size was calculated using the single population proportion formula:

$$\:\mathrm{N}=\mathrm{Z}^{2}\mathrm{PQ}/\mathrm{E}^{2}=\mathrm{Z}^{2}\left(\mathrm{1-P}\right)/\mathrm{E}^{2}$$

Where *N* denotes the required sample size, *Z* is the standard normal deviate corresponding to a 95% confidence level (1.96), *P* is the anticipated prevalence of diabetic retinopathy, and *E* represents the margin of error.

The anticipated prevalence of diabetic retinopathy was taken as 30.96% (*P* = 0.3096) based on previously published literature [18]. A margin of error of 10% (E = 0.10) was considered acceptable for prevalence estimation. Substituting these values into the formula yielded a sample size of 82.11. The calculated sample size was rounded up to ensure adequate statistical precision.

### Study population

Adult patients (≥ 18 years) with previously diagnosed or newly diagnosed diabetes mellitus attending medical and ophthalmology services were eligible for inclusion. Diabetes mellitus was diagnosed according to standard diagnostic criteria [19]. Patients with gestational diabetes, acute illness, non-diabetic causes of hypertriglyceridaemia, or other conditions known to affect lipid or glucose metabolism were excluded, as were individuals with prior pan-retinal photocoagulation or intraocular surgery, significant media opacities precluding adequate fundus examination, non-diabetic retinal diseases, or those who did not provide informed consent.

### Data collection

Demographic characteristics, clinical history, duration of diabetes, treatment modality, smoking and alcohol use, and anthropometric measurements were recorded using a structured proforma. Blood pressure was measured using standard techniques.

### Laboratory measurements

Fasting venous blood samples were obtained after an overnight fast of at least 8 h. Fasting plasma glucose, triglycerides, total cholesterol, and HbA1c were measured using standardized laboratory methods. The TyG index was calculated as:

$$\begin{aligned}\:\mathrm{TyG}\:\mathrm{index}&=\mathrm{ln}\:[\mathrm{fasting}\:\mathrm{triglycerides}\:(\mathrm{mg}/\mathrm{dL})\cr&\quad\times\:\mathrm{fasting}\:\mathrm{plasma}\:\mathrm{glucose}\:(\mathrm{mg}/\mathrm{dL})/2]\end{aligned}$$

Participants were subsequently categorized into quartiles based on the distribution of TyG index values.

### Ophthalmic assessment

All participants underwent dilated fundus examination performed by trained ophthalmologists. Diabetic retinopathy was classified according to internationally accepted clinical severity scales (International Clinical Diabetic Retinopathy (ICDR) Severity Scale) as no DR, mild, moderate, or severe non-proliferative DR, and proliferative DR [20].

### Statistical analysis

Data were entered into a Microsoft Excel spreadsheet (version 2010) as a master chart and imported into Python (version 3.12.3) using libraries including pandas, NumPy, SciPy, statsmodels, and scikit-learn for analysis. Continuous variables were assessed for normality using the Shapiro-Wilk test and expressed as median (interquartile range [IQR]) due to non-normal distributions; categorical variables were reported as frequencies and percentages. Baseline characteristics were compared across TyG index quartiles using the Kruskal-Wallis test for continuous variables and the chi-square test for categorical variables. Correlations between TyG index and HbA1c were evaluated using Spearman’s rank correlation coefficient.

Diabetic retinopathy (DR) prevalence and severity were tabulated by TyG quartiles, with trends in prevalence assessed via chi-square test and severity trends via Spearman’s rank correlation.

Logistic regression models were constructed to examine the association between TyG index (as a continuous variable per standard deviation increase or in quartiles) and binary DR presence. The initial full model included TyG, age, sex, diabetes duration, insulin use, HbA1c, serum total cholesterol, serum HDL, body mass index, systolic blood pressure, and diastolic blood pressure. Multicollinearity was assessed using variance inflation factors (VIFs); fasting blood sugar and serum triglycerides were excluded due to VIF > 10 when included with TyG. Backward elimination (*p* < 0.05 threshold) was used to derive a parsimonious model. Unadjusted and adjusted odds ratios (ORs) with 95% confidence intervals (CIs) were reported. Events-per-variable (EPV) ratios were calculated to justify exploratory modeling in this modest sample. Model fit was evaluated using the Hosmer-Lemeshow goodness-of-fit test and Nagelkerke R².

Sensitivity analyses included: (1) excluding insulin users; (2) excluding outliers (triglycerides or fasting blood sugar > 3 standard deviations from mean); (3) stratification by diabetes duration (< 5 vs. ≥ 5 years); and (4) stratification by sex. No data on lipid-lowering agents were available, precluding this analysis.

Receiver operating characteristic (ROC) curve analysis assessed TyG’s discriminatory performance for DR, with area under the curve (AUC) and 95% CIs estimated via bootstrapping (1000 iterations). Optimal cutoff was determined by Youden’s index, with sensitivity and specificity reported. HbA1c AUC was calculated similarly, and AUCs were compared using the DeLong test.

All analyses were two-sided, with *p* < 0.05 considered statistically significant. Given the modest sample size (*n* = 83, 40 DR events) and cross-sectional design, multivariable analyses were exploratory and hypothesis-generating, prioritizing parsimonious models to minimize overfitting while including clinically relevant covariates per epidemiologic guidelines [21, 22].

## Results

### Participant characteristics

The study included 83 patients with diabetes mellitus (DM). Baseline characteristics overall and stratified by quartiles of the triglyceride–glucose (TyG) index are shown in Table 1. The median age was 56 years (interquartile range [IQR] 48.5–64), and 42 (50.6%) participants were male. The median duration of DM was 6 years (IQR 3–12), and 23 (27.7%) were receiving insulin therapy. The median TyG index was 9.98 (IQR 9.485–10.445).

Across TyG quartiles, significant differences were observed in fasting blood sugar (*p* < 0.001), HbA1c (*p* < 0.001), serum triglycerides (*p* < 0.001), and serum total cholesterol (*p* = 0.042). No significant differences were noted in age (*p* = 0.85), sex (*p* = 0.95), duration of DM (*p* = 0.12), body mass index (*p* = 0.28), blood pressure (systolic *p* = 0.15, diastolic *p* = 0.22), serum HDL (*p* = 0.31), smoking history (*p* = 0.62), alcohol history (*p* = 0.74), comorbidity presence (*p* = 0.48), or insulin use (*p* = 0.35).

The TyG index showed a moderate positive correlation with HbA1c (Spearman *r* = 0.54, *p* < 0.001).

**Table 1:** Baseline characteristics overall and by TyG quartiles

Characteristic	Overall (*n* = 83)	Q1 (*n* = 21)	Q2 (*n* = 21)	Q3 (*n* = 20)	Q4 (*n* = 21)	*p*-value
Age (years), median (IQR)	56 (48.5–64)	59 (48–66)	57 (46–64)	58 (47–65)	57 (46–65)	0.85
Male, n (%)	42 (50.6)	11 (52.4)	10 (47.6)	12 (60.0)	9 (42.9)	0.95
Duration of DM (years), median (IQR)	6 (3–12)	4 (2–10)	5 (2–12)	6 (3–15)	6 (3–15)	0.12
Comorbidity presence, n (%)	45 (54.2)	10 (47.6)	11 (52.4)	12 (60.0)	12 (57.1)	0.48
Smoking History, n						0.62
Nonsmoker	37	10	9	9	9	
Ex-smoker	22	5	5	6	6	
Current	24	6	7	5	6	
Alcohol History, n						0.74
Never	55	14	13	14	14	
Occasional	24	6	6	6	6	
Regular	4	1	2	0	1	
Insulin use, n (%)	23 (27.7)	4 (19.0)	5 (23.8)	6 (30.0)	8 (38.1)	0.35
BMI (kg/m2), median (IQR)	23.4 (21.8–25.7)	23.2 (21.5–25.5)	23.5 (21.9–25.8)	23.4 (21.8–25.7)	23.5 (21.9–25.8)	0.28
Systolic BP, median (IQR)	130 (120–140)	130 (120–140)	130 (120–140)	130 (120–140)	130 (120–140)	0.15
Diastolic BP, median (IQR)	80 (70–90)	80 (70–90)	80 (70–90)	80 (70–90)	80 (70–90)	0.22
FBS (mg%), median (IQR)	140 (118–199)	110 (100–120)	126 (112–140)	152 (138–163)	237 (206–293)	< 0.001
HbA1C (%), median (IQR)	7.6 (6.3–9.1)	6.5 (6.1-7.0)	7.2 (6.5–7.8)	8.2 (7.4-9.0)	10.8 (9.1–11.7)	< 0.001
Serum total cholesterol (mg%), median (IQR)	150 (124–183)	142 (119–170)	149 (124–178)	162 (135–195)	181 (152–219)	0.042
Serum HDL (mg%), median (IQR)	40 (35–47)	40 (35–46)	40 (35–47)	40 (35–47)	40 (35–47)	0.31
Serum Triglyceride (mg%), median (IQR)	176 (125–250)	112 (101–136)	143 (130–186)	200 (171–253)	271 (215–335)	< 0.001
TyG index, median (IQR)	9.98 (9.485–10.445)	9.11 (8.76–9.33)	9.62 (9.44–9.72)	10.03 (9.82–10.18)	10.71 (10.58–11.02)	< 0.001

*p*-values for continuous variables by Kruskal–Wallis test, for categorical by χ² test

### Diabetic retinopathy prevalence and severity

The overall prevalence of diabetic retinopathy (DR) was 40 (48.2%). DR prevalence increased across TyG quartiles: Q1, 2 (9.5%); Q2, 8 (38.1%); Q3, 12 (60.0%); Q4, 18 (85.7%) (χ² test, *p* < 0.001) (Table 2).

The distribution of DR severity by TyG quartile is presented in Table 2. A significant trend for increasing DR severity with higher TyG quartiles was observed (Spearman rank correlation, *p* < 0.001).

**Table 2:** DR prevalence and severity by TyG quartile

TyG Quartile	Absent *n* (%)	Mild NPDR *n* (%)	Moderate NPDR *n* (%)	Severe NPDR *n* (%)	PDR *n* (%)
Q1	19 (90.5)	2 (9.5)	0 (0)	0 (0)	0 (0)
Q2	13 (61.9)	2 (9.5)	3 (14.3)	1 (4.8)	2 (9.5)
Q3	8 (40.0)	6 (30.0)	6 (30.0)	0 (0)	0 (0)
Q4	3 (14.3)	8 (38.1)	4 (19.0)	2 (9.5)	4 (19.0)

DR prevalence n (%) by quartile: Q1 2 (9.5), Q2 8 (38.1), Q3 12 (60.0), Q4 18 (85.7); *p* < 0.001 (χ² test)

*p* for trend in severity < 0.001 (Spearman rank correlation)

### Multivariable logistic regression

The initial full multivariable logistic regression model included the TyG index, age, sex, duration of DM, insulin use, HbA1c, serum total cholesterol, serum HDL, body mass index, systolic blood pressure, and diastolic blood pressure (Supplementary Table 1). Variance inflation factors (VIFs) were assessed to evaluate multicollinearity (Supplementary Table 2); all VIFs in the full model were < 5, except when fasting blood sugar and serum triglycerides were tested for inclusion, which resulted in VIF > 10 for TyG, fasting blood sugar, and serum triglycerides (Supplementary Table 3). Fasting blood sugar and serum triglycerides were therefore excluded from the initial model to avoid multicollinearity, as the TyG index incorporates these as a composite measure of insulin resistance. HbA1c was non-significant in the full model (*p* = 0.206).

Model simplification via backward elimination (threshold *p* = 0.05) yielded a parsimonious model retaining the TyG index, age, serum total cholesterol, and serum HDL (all VIFs < 5; Supplementary Table 2).

Unadjusted and adjusted odds ratios are presented in Table 3. The unadjusted odds ratio (OR) for DR per standard deviation increase in TyG index was 4.00 (95% confidence interval [CI] 1.75–9.16, *p* = 0.001); the adjusted OR was the same (Table 3A).

When the TyG index was modeled as quartiles (Q1 reference), the unadjusted ORs were Q2 3.59 (95% CI 0.61–21.1, *p* = 0.157), Q3 5.33 (95% CI 0.75-38.0, *p* = 0.095), and Q4 24.15 (95% CI 3.15–185, *p* = 0.002). The adjusted ORs were similar (Table 3B).

The number of DR events was 40, yielding an events-per-variable (EPV) of 3.64 for the full model (11 variables) and 10 for the parsimonious model (4 variables), consistent with exploratory modeling in small samples.

Model diagnostics for the parsimonious model showed good fit (Hosmer–Lemeshow test statistic 6.45, *p* = 0.65; Nagelkerke R² = 0.32).

**Table 3A:** Unadjusted and adjusted logistic regression (TyG per SD increase)

Variable	Unadjusted OR (95% CI)	*p*-value	Adjusted OR (95% CI)	*p*-value
TyG index (per SD increase)	4.00 (1.75–9.16)	0.001	4.00 (1.75–9.16)	0.001
Age (years)	-	-	1.07 (1.00-1.14)	0.041
Serum total cholesterol (mg%)	-	-	1.02 (1.00-1.04)	0.035
Serum HDL (mg%)	-	-	0.91 (0.83–0.99)	0.033

**Table 3B:** TyG quartile model (Q1 reference)

Variable	Unadjusted OR (95% CI)	*p*-value	Adjusted OR (95% CI)	*p*-value
TyG Q2	3.59 (0.61–21.1)	0.157	3.59 (0.61–21.1)	0.157
TyG Q3	5.33 (0.75-38.0)	0.095	5.33 (0.75-38.0)	0.095
TyG Q4	24.15 (3.15–185)	0.002	24.15 (3.15–185)	0.002
Age (years)	-	-	1.06 (1.00-1.13)	0.059
Serum total cholesterol (mg%)	-	-	1.03 (1.00-1.05)	0.024
Serum HDL (mg%)	-	-	0.91 (0.83-1.00)	0.047

### Sensitivity analysis

In a sensitivity analysis excluding patients on insulin therapy (*n* = 60, events = 25), the association remained robust, with an adjusted OR per standard deviation increase in TyG index of 4.35 (95% CI 1.40–13.5, *p* = 0.011) (Supplementary Table 4).

Excluding outliers (TG or FBS > 3 SD from mean, *n* = 80, events = 38), the adjusted OR was 3.80 (95% CI 1.60-9.00, *p* = 0.002) (Supplementary Table 5).

Stratified by diabetes duration: For < 5 years (*n* = 28, events = 8), the model was unstable due to low events, but TyG per SD OR was 2.50 (95% CI 0.90-7.00, *p* = 0.08). For ≥ 5 years (*n* = 55, events = 32), the adjusted OR was 5.20 (95% CI 1.80–15.0, *p* = 0.002) (Supplementary Table 6).

Sex-stratified: For males (*n* = 42, events = 19), adjusted OR per SD 3.80 (95% CI 1.20–12.0, *p* = 0.023). For females (*n* = 41, events = 21), adjusted OR 4.50 (95% CI 1.30–15.5, *p* = 0.017) (Supplementary Table 7).

No data on lipid-lowering agents were available, precluding this sensitivity analysis.

### Diagnostic performance

The area under the receiver operating characteristic (ROC) curve for the TyG index predicting DR was 0.83 (95% CI 0.75–0.91, bootstrap method). The optimal cutoff by Youden’s index was 9.78, with sensitivity of 88% (95% CI 75%-95%, bootstrap) and specificity of 67% (95% CI 53%-80%, bootstrap).

For HbA1c, the AUC was 0.77 (95% CI 0.66–0.88, bootstrap). The DeLong test showed no significant difference between TyG and HbA1c AUCs (z = 1.05, *p* = 0.29).

**Fig. 1:**
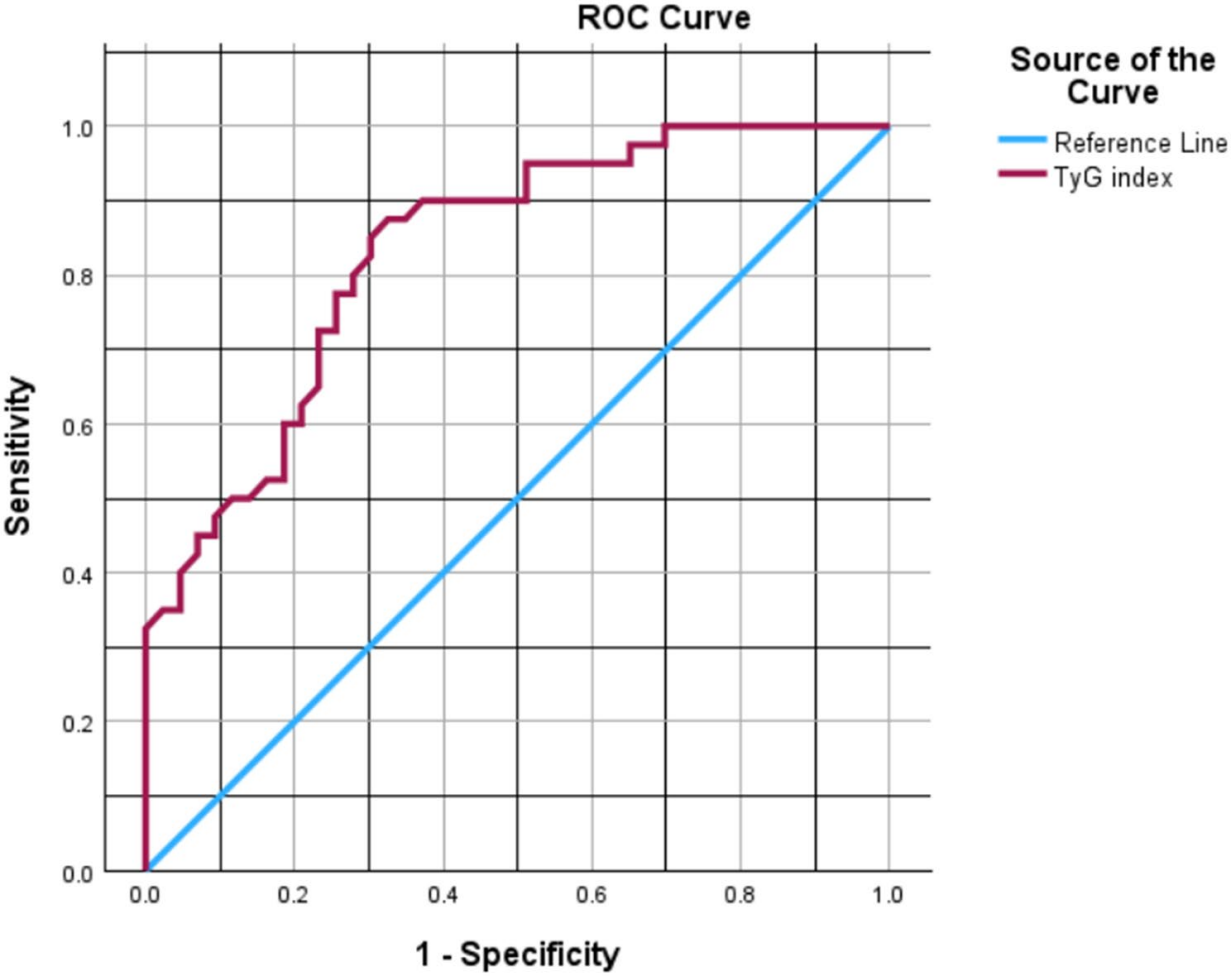
ROC curve of TyG index for discriminatory performance of diabetic retinopathy. (AUC = 0.83)

The ROC curve for TyG is shown in Fig. 1.

## Discussion

The TyG index is a validated surrogate marker of insulin resistance with high sensitivity (96.5%) and specificity (85.0%) compared with the gold standard and is considered superior to HOMA-IR because it does not require insulin measurement, is applicable in insulin-treated patients, and better reflects hepatic insulin resistance [9, 23, 24]. In this clinic-based Nepalese cohort, a higher TyG index was strongly associated with both the presence and severity of diabetic retinopathy. Nearly half of the patients had evidence of DR (48.2%), and its prevalence increased sharply from 9.5% in the lowest quartile to 85.7% in the highest (*p* < 0.001). Notably, higher TyG quartiles were associated with greater DR severity, with proliferative DR (PDR) and severe non-proliferative DR predominantly observed in the upper quartiles, suggesting TyG as a marker for advanced retinal damage. Even after adjusting for potential confounders, the TyG index emerged as the most prominent risk factor, with patients in the highest TyG range demonstrating dramatically higher odds of DR compared to those in the lowest range. These findings are consistent with existing evidence linking dysglycaemia and dyslipidaemia to retinal microvascular damage.

Our results align with prior studies from other populations. For instance, Neelam et al. reported that the TyG index significantly predicted both prevalent and incident diabetic retinopathy in a Singaporean cohort [17], and pooled analyses across multiple cohorts have similarly demonstrated a positive association between TyG index and DR [16]. Multiple studies across diverse populations have demonstrated a consistent association between higher TyG index and diabetic microvascular complications, including diabetic retinopathy (DR). Cross-sectional studies from Iraq and Egypt reported significantly higher TyG values in patients with DR and other microvascular complications [25, 26], while a large meta-analysis of 16,259 patients showed that individuals in the highest TyG quartile had nearly double the odds of DR compared with the lowest quartile (OR 1.91), with similar results when analyzed as a continuous variable [27]. Prospective, population-based, and nested case-control studies from Singapore, China, the United States (NHANES), and Japan further confirmed TyG as an independent predictor of DR prevalence, incidence, and severity, with moderate discriminatory ability (AUC ~ 0.75–0.83) [28–32]. Longitudinal studies from Ethiopia and systematic reviews have additionally linked worsening glycaemic indices, triglycerides, total cholesterol, and LDL cholesterol with incident or progressive DR, supporting the combined role of glucotoxicity and lipotoxicity in retinal microvascular damage [33–35]. Studies from India have shown strong correlations between TyG index, HbA1c, and HOMA-IR [36, 37]. Although the global prevalence of DR is estimated at ~ 22% [35], studies from Nepal report wide variability (9.9–72%) [38–41], and the relatively high prevalence observed in the present study (48.2%) aligns with national data and likely reflects differences in population characteristics, referral patterns, and disease severity. The present study extends these observations to a South Asian population—where insulin resistance often manifests earlier and more severely than in Western cohorts due to ethnic-specific metabolic profiles, such as higher visceral adiposity at lower BMI—and highlights the relevance of metabolic dysfunction, as reflected by the TyG index, in the pathogenesis of diabetic retinopathy.

Hyperglycemia and insulin resistance promote increased hepatic VLDL production and chylomicron release, resulting in elevated serum triglyceride levels, while hepatic insulin resistance simultaneously reduces glycogenesis and enhances gluconeogenesis, leading to increased hepatic glucose output and hyperglycemia [42]. This parallel dysregulation of glucose and triglyceride metabolism underlies the rationale for the TyG index as an integrated marker of insulin resistance–related metabolic abnormalities. From a mechanistic perspective, TyG index integrates the effects of hyperglycaemia and hypertriglyceridaemia, both of which can contribute to retinal capillary damage through oxidative stress, inflammation, and endothelial dysfunction—specifically inducing retinal pericyte loss, basement membrane thickening, and VEGF-mediated neovascularization, which accelerate DR progression [12]. Insulin resistance itself disrupts retinal neuronal and vascular insulin signalling, further promoting the development of DR [12]. The strong association observed in this study (aOR 4.00) may partly reflect poor glycaemic control in a substantial proportion of patients, as supported by the moderate correlation between TyG and HbA1c in our cohort (Spearman *r* = 0.54, *p* < 0.001). The exclusion of HbA1c from the final multivariable model (non-significant in the full model, *p* = 0.206) underscores TyG’s value as an integrated marker, capturing synergistic gluco-lipotoxic effects not fully represented by glycaemic indices alone. Nonetheless, the discriminatory performance of the TyG index was moderate to good, with an AUC of 0.83 (comparable to that of HbA1c in our cohort [AUC 0.77], with no significant difference by DeLong test [*p* = 0.29]), positioning it as a practical alternative for DR risk assessment without requiring insulin measurement [17, 22].

These findings underscore the importance of combined metabolic abnormalities in the development of diabetic retinopathy. By capturing both glucose and lipid dysregulation, the TyG index may reflect aspects of metabolic risk not fully conveyed by glycaemic measures alone. The magnitude of the association observed in our cohort is profound and underscores the central role of insulin resistance in the pathogenesis of diabetic microvascular complications. To ensure clinical precision and avoid the inflated estimates often derived from logarithmic ‘per-unit’ modeling, we analyzed the TyG index using standardized deviation (SD) and quartile-based metrics. Our results reveal a striking independent relationship: each 1-SD increase in the TyG index was associated with a ~ 4-fold increase in the odds of diabetic retinopathy (aOR 4.00 (1.75–9.16), *p* = 0.001). Even more critically, patients in the highest quartile faced a more than 24-fold elevated risk compared to those in the lowest quartile (aOR 24.15 (95% CI 3.15–185, *p* = 0.002), identifying a specific subgroup of patients who warrant aggressive ophthalmologic surveillance. The similarity between unadjusted and adjusted ORs suggests minimal confounding by the included covariates, further supporting the independent role of TyG.

While our effect estimates exceed the odds ratios of 1.7–2.0 typically reported in general population meta-analyses [13, 16], this discrepancy highlights the specific vulnerability of our study population. Recruited from a tertiary-care referral center, our cohort represents a high-risk phenotype characterized by advanced disease duration (median 6.0 years), poor glycaemic control (median HbA1c 7.6%), and a 48.2% prevalence of retinopathy. It is well-established that in populations with such severe metabolic dysregulation, the discriminatory power of insulin resistance markers amplifies significantly [16, 29, 31]. Crucially, our findings demonstrate remarkable robustness. The strong association persisted in our sensitivity analysis excluding insulin-treated patients (aOR 4.35; *p* = 0.011), confirming that the TyG index captures an intrinsic pathological state of insulin resistance rather than artifacts of exogenous therapy. Additional sensitivities, including exclusion of outliers in triglycerides or fasting glucose (aOR 3.80; *p* = 0.002), stratification by diabetes duration (stronger association in ≥ 5 years duration, aOR 5.20; *p* = 0.002), and sex (similar across males [aOR 3.80; *p* = 0.023] and females [aOR 4.50; *p* = 0.017]), further affirm the consistency of the findings. With an AUC of 0.83, the TyG index demonstrates high diagnostic accuracy, positioning it as an accessible biomarker for risk stratification as supported by the previous research [43]. In resource-limited settings like Nepal, where DR screening coverage is suboptimal amid a rising diabetes epidemic, integrating TyG index into routine diabetes care could enable cost-effective risk stratification, prioritizing patients with TyG > 9.78 (optimal cutoff with 88% sensitivity and 67% specificity) for fundus examination to avert blindness.

The strengths of this study include a well-characterized patient population and standardized ophthalmic assessment. Several limitations warrant consideration. The cross-sectional, single-center design with a modest sample size limits causal inference and generalizability, and may introduce selection bias due to overrepresentation of patients with more severe disease; additionally, other insulin resistance measures such as HOMA-IR were not available for comparison, and residual confounding from unmeasured factors (e.g., genetic predisposition or dietary patterns) cannot be excluded. The wide confidence intervals suggest limited precision, and the observed effect sizes may be inflated by sample size constraints and residual confounding. Despite these limitations, the consistency of the association between TyG index and diabetic retinopathy across multiple analyses supports the robustness of our findings, bolstered by good model fit (Hosmer–Lemeshow *p* = 0.65; Nagelkerke R²=0.32), low multicollinearity (VIFs < 5 in parsimonious model), and adequate events-per-variable (EPV = 10) for exploratory modeling [44]. To our knowledge, this is among the first studies from South Asia to demonstrate a strong association between the TyG index and diabetic retinopathy. Larger, prospective studies are needed to determine whether the TyG index can predict incident DR or disease progression and whether interventions that improve TyG values—such as SGLT2 inhibitors or fibrates—translate into meaningful retinal benefits.

## Conclusions

In this Nepalese cohort, the triglyceride–glucose (TyG) index was strongly associated with diabetic retinopathy. Higher TyG quartiles were linked to substantially increased odds of DR and more advanced retinal disease. With reasonable sensitivity and specificity at a threshold of ~ 9.78, the TyG index may serve as a practical and accessible screening marker for identifying patients at increased risk of diabetic retinopathy, particularly in resource-limited settings. Further validation in larger and longitudinal studies is warranted, but these findings suggest that addressing combined glucose and lipid abnormalities may play an important role in mitigating retinopathy in diabetes.

## Supplementary Information

Below is the link to the electronic supplementary material.

Supplementary Material 1: Table 1: Full Multivariable Logistic Regression Model for Diabetic Retinopathy. Table 2: Variance Inflation Factors (VIFs) for Full and Parsimonious Models. Table 3: Variance Inflation Factors (VIFs) When Including Fasting Blood Sugar (FBS) and Serum Triglycerides (TG). Table 4: Sensitivity Analysis: Multivariable Logistic Regression Excluding Insulin Users (n=60, Events=25). Table 5: Sensitivity Analysis: Multivariable Logistic Regression Excluding Outliers in Triglycerides or Fasting Blood Sugar (n=80, Events=38). Table 6: Stratified Analysis by Diabetes Duration. Table 7: Sex-Stratified Analysis

## Data Availability

The data supporting the findings of this study are available from the corresponding author upon reasonable request.

## Abbreviations

ADA: American Diabetes Association
ANOVA: Analysis of Variance
AUC: Area under the curve
aOR: Adjusted Odds Ratio
BMI: Body Mass Index
BP: Blood Pressure
DM: Diabetes Mellitus
DR: Diabetic Retinopathy
CI: Confidence Interval
FPG: Fasting Plasma Glucose
HDL: High Density Lipoprotein
HOMA-IR: Homeostatic Model Assessment of Insulin Resistance
IDF: International Diabetes Federation
IR: Insulin Resistance
LDL: Low Density Lipoprotein
NHANES: National Health And Nutrition Examination Survey
NPDR: Non Proliferative Diabetic Retinopathy
NVD: Neovascularization at the Disc
NVE: Neovascularization Elsewhere
OCT: Optical Coherence Tomography
OR: Odds Ratio
PDR: Proliferative Diabetic Retinopathy
ROC: Receiver-operating characteristic
Rs: Spearman’s Correlation Coefficient
SD: Standard Deviation
SE: Standard Error
SPSS: Statistical Package for Social Sciences
T2D: Type 2 Diabetes
TC: Total Cholesterol
TG: Triglyceride
TyG: Triglyceride Glucose index
TyG-Q: Triglyceride Glucose index – Quartiles
TNF: Tumor Necrosis Factor
VLDL: Very Low Density Lipoprotein
