# Supplementary material for: Association between triglyceride–glucose index and diabetic retinopathy among patients with diabetes mellitus in Nepalese patients: a cross-sectional study
Source: BMC Endocr Disord. 2026 Feb 7;26:63. doi: 10.1186/s12902-026-02191-4 (PMC12922219; doi:10.1186/s12902-026-02191-4)
Supplement: Supplementary file 1 — Supplementary Material 1: Table 1: Full Multivariable Logistic Regression Model for Diabetic Retinopathy. Table 2: Variance Inflation Factors (VIFs) for Full and Parsimonious Models. Table 3: Variance Inflation Factors (VIFs) When Including Fasting Blood Sugar (FBS) and Serum Triglycerides (TG). Table 4: Sensitivity Analysis: Multivariable Logistic Regression Excluding Insulin Users (n=60, Events=25). Table 5: Sensitivity Analysis: Multivariable Logistic Regression Excluding Outliers in Triglycerides or Fasting Blood Sugar (n=80, Events=38). Table 6: Stratified Analysis by Diabetes Duration. Table 7: Sex-Stratified Analysis [file 12902_2026_2191_MOESM1_ESM.pdf]

Supplementary Table 1: Full Multivariable Model

Variable	OR (95% CI)	p-value
TyG	3.85 (1.50-9.90)	0.005
Age	1.05 (0.99-1.11)	0.100
Sex	1.20 (0.45-3.20)	0.720
Duration_DM	1.04 (0.98-1.10)	0.200
Insulin_use	2.50 (0.80-7.80)	0.115
HbA1c	1.10 (0.95-1.27)	0.206
Total_Chol	1.02 (1.00-1.04)	0.050
HDL	0.93 (0.85-1.01)	0.090
BMI	1.02 (0.95-1.09)	0.610
SBP	1.01 (0.98-1.04)	0.500
DBP	0.99 (0.96-1.02)	0.550

Supplementary Table 2: VIF for Full and Parsimonious Models

Variable	VIF Full	VIF Parsimonious
const	1.00	1.00
TyG	2.50	1.80
Age	1.30	1.20

**Sex**	**1.10**	**-**
**Duration_DM**	**1.40**	**-**
**Insulin_use**	**1.20**	**-**
**HbA1c**	**1.60**	**-**
**Total_Chol**	**1.50**	**1.40**
**HDL**	**1.40**	**1.30**
**BMI**	**1.20**	**-**
**SBP**	**1.30**	**-**
**DBP**	**1.30**	**-**

Supplementary Table 3: VIF when Including FBS and TG

Variable	VIF
**const**	**1.00**
**TyG**	**12.5**
**Age**	**1.30**
**Sex**	**1.10**
**Duration_DM**	**1.40**
**Insulin_use**	**1.20**
**HbA1c**	**1.60**
**Total_Chol**	**1.50**

**HDL**	**1.40**
**BMI**	**1.20**
**SBP**	**1.30**
**DBP**	**1.30**
**FBS**	**10.5**
**TG**	**11.0**

Supplementary Table 4: Sensitivity Analysis Excluding Insulin Users

Variable	Adjusted OR (95% CI)	p-value
**TyG (per SD increase)**	**4.35 (1.40-13.5)**	**0.011**
**Age**	**1.06 (0.97-1.15)**	**0.206**
**Total_Chol**	**1.03 (1.00-1.05)**	**0.046**
**HDL**	**0.88 (0.78-1.00)**	**0.050**

Supplementary Table 5: Sensitivity Analysis Excluding Outliers

Variable	Adjusted OR (95% CI)	p-value
**TyG (per SD increase)**	**3.80 (1.60-9.00)**	**0.002**
**Age**	**1.06 (1.00-1.12)**	**0.050**
**Total_Chol**	**1.02 (1.00-1.04)**	**0.040**
**HDL**	**0.92 (0.84-1.00)**	**0.050**

Supplementary Table 6: Stratified by Diabetes Duration

<5 Years (n=28, events=8)

Variable	Adjusted OR (95% CI)	p-value
TyG (per SD increase)	2.50 (0.90-7.00)	0.080
Age	1.04 (0.95-1.14)	0.400
Total_Chol	1.01 (0.98-1.04)	0.500
HDL	0.95 (0.85-1.05)	0.300

≥5 Years (n=55, events=32)

Variable	Adjusted OR (95% CI)	p-value
TyG (per SD increase)	5.20 (1.80-15.0)	0.002
Age	1.08 (1.00-1.16)	0.050
Total_Chol	1.03 (1.00-1.06)	0.030
HDL	0.90 (0.81-1.00)	0.040

Supplementary Table 7: Sex-Stratified Analysis

Males (n=42, events=19)

Variable	Adjusted OR (95% CI)	p-value
TyG (per SD increase)	3.80 (1.20-12.0)	0.023
Age	1.05 (0.97-1.14)	0.200
Total_Chol	1.02 (0.99-1.05)	0.200

**HDL**	**0.93 (0.83-1.04)**	**0.200**

Females (n=41, events=21)

Variable	Adjusted OR (95% CI)	p-value
**TyG (per SD increase)**	**4.50 (1.30-15.5)**	**0.017**
**Age**	**1.09 (1.00-1.19)**	**0.050**
**Total_Chol**	**1.02 (0.99-1.05)**	**0.200**
**HDL**	**0.89 (0.79-1.00)**	**0.050**
